# Generation of hydroxyl radicals and singlet oxygen during oxidation of rhododendrol and rhododendrol-catechol

**DOI:** 10.3164/jcbn.16-38

**Published:** 2016-10-05

**Authors:** Akimitsu Miyaji, Yu Gabe, Masahiro Kohno, Toshihide Baba

**Affiliations:** 1 Department of Environmental Chemistry and Engineering, Tokyo Institute of Technology, 4259-G1-14, Nagatsuta-cho, Midori-ku, Yokohama 226-8502, Japan; 2 Biological Science Laboratories, Kao Corporation, 2606 Akabane, Ichikai-machi, Haga-gun, Tochigi 321-3497, Japan; 3 Department of Bioengineering, Tokyo Institute of Technology, 4259-G1-25, Nagatsuta-cho, Midori-ku, Yokohama 226-8502, Japan

**Keywords:** autoxidation, hydroxyl radical, rhododendrol, singlet oxygen, tyrosinase

## Abstract

The generation of hydroxyl radicals and singlet oxygen during the oxidation of 4-(4-hydroxyphenyl)-2-butanol (rhododendrol) and 4-(3,4-dihydroxyphenyl)-2-butanol (rhododendrol-catechol) with mushroom tyrosinase in a phosphate buffer (pH 7.4) was examined as the model for the reactive oxygen species generation via the two rhododendrol compounds in melanocytes. The reaction was performed in the presence of 5,5-dimethyl-1-pyrroline-*N*-oxide (DMPO) spin trap reagents for hydroxyl radical or 2,2,6,6-tetramethyl-4-piperidone (4-oxo-TEMP), an acceptor of singlet oxygen, and their electron spin resonances were measured. An increase in the electron spin resonances signal attributable to the adduct of DMPO reacting with the hydroxyl radical and that of 4-oxo-TEMP reacting with singlet oxygen was observed during the tyrosinase-catalyzed oxidation of rhododendrol and rhododendrol-catechol, indicating the generation of hydroxyl radical and singlet oxygen. Moreover, hydroxyl radical generation was also observed in the autoxidation of rhododendrol-catechol. We show that generation of intermediates during tyrosinase-catalyzed oxidation of rhododendrol enhances oxidative stress in melanocytes.

## Introduction

The generation of reactive oxygen species (ROS) during tyrosinase-catalyzed oxidation reactions has been reported previously.^(1)^ The generation of hydrogen peroxide and superoxide anions during the oxidation of l-tyrosine in the presence of tyrosinase was observed using a chemiluminescence probe.^(2)^ The generation of hydroxyl radicals during the oxidation of l-tyrosine and l-dopa in the presence of tyrosinase was confirmed using an electron spin resonance (ESR) spin trapping reagent, 5,5-dimethyl-1-pyrroline-*N*-oxide (DMPO).^(3)^ These results suggest that ROS generation occurs in melanocytes, the cells synthesizing melanin in which tyrosinase operates.

Once the balance of ROS generation and the antioxidant capacity in melanocytes is disturbed, excess ROS in melanocytes causes a decrease in the antioxidant capacity due to oxidative stress.^(4)^ ROS damage DNA, proteins, and lipids in cells and, thus, are responsible for the pathogenesis of numerous diseases. Oxidative stress is thought to be involved in the development of skin diseases such as melanoma.^(5)^

**Fig. 1:**
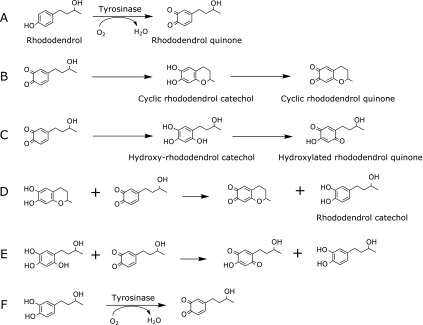
Rhododendrol oxidation.

Recently, those who used lightening and whitening cosmetics containing 4-(4-hydroxyphenyl)-2-butanol (rhododendrol) suffered from leukoderma,^(6)^ a cutaneous condition with localized loss of pigmentation. Rhododendrol can cause tyrosinase-related toxicity in cells.^(7,8)^ Rhododendrol is converted to catechol- and *o*-quinone derivatives.^(9,10)^ Reduction of melanin synthesis in melanocytes by rhododendrol seems to be due to competition between rhododendrol oxidation and l-tyrosine oxidation at tyrosinase active sites. 4-(3-hydroxybutyl)-1,2-benzoquinone (rhododendrol quinone), 4-(3,4-dihydroxyphenyl)-2-butanol (rhododendrol-catechol), 6,7-dihydroxy-2-methylchromane (Cyclic rhododendrol-catechol), and 5-(3-hydroxybutyl)-benzene-1,2,4-triol (hydroxyl rhododendrol-catechol) have been identified as reaction products.^(9)^ Rhododendrol-quionone is the product of tyrosinase-catalyzed rhododendrol oxidation as shown in Fig. 1A. Cyclic rhododendrol-catechol is produced by the cyclization of rhododendrol-quinone as shown in Fig. 1B, which is similar to cyclization of dopaquionone to leukodopachrome in the synthesis of melanin from l-tyrosine.^(11)^ Hydroxyl rhododendrol-catechol is probably produced by hydroxylation of rhododendrol-quinone as shown in Fig. 1C. Rhododendrol-catechol may be produced by the reaction of rhododendrol-quinone with other catechol-derivatives as shown in Fig. 1D and E. This latter reaction is predicted based on l-dopa generation in tyrosinase-catalyzed l-tyrosine oxidation in melanogenesis.^(11)^ Rhododendrol-catechol is also oxidized by tyrosinase as shown in Fig. 1F. Rhododenrol-catechol shows higher toxicity in cells than rhododendrol.^(7,8)^

One of the reasons for leukoderma caused by rhododendrol is thought to be oxidative stress induced by tyrosinase-catalyzed oxidation.^(12)^ In fact, rhododendrol increased the level of intracellular ROS and hydrogen peroxide in melanoma cells. The generation of hydroxyl radicals was observed in a mixture of rhododendrol, tyrosinase, and hydrogen peroxide, and the generation was enhanced by irradiation with 1,000 J/m^2^ UV-B (ultraviolet light with 280–315 nm-wavelength).^(13)^ Further, ROS generation was detected during rhododendrol oxidation by tyrosinase.^(14)^ Hence, in addition to hydrogen peroxide, hydroxyl radicals and singlet oxygen (^1^O_2_) are strong candidates for the onset of leukoderma caused by rhododendrol.

The aim of this study is to study the generation of hydroxyl radicals and ^1^O_2_ during the oxidation of rhododendrol and rhododendrol-catechol. We measured the amount of ROS present during the oxidation that occurred at a pH of ~7 so that the oxidative stress caused by the imbalance of ROS generation and antioxidant capacity in biological systems could be investigated. We applied the electron spin resonance (ESR) spin-trapping method to measure the generation of hydroxyl radicals and ^1^O_2_ during rhododendrol oxidation. A hydrophilic spin-trapping probe working at a pH of ~7 was used under conditions needed for carrying out enzymatic reactions. Thus, 5,5-dimethyl-1-pyrroline-*N*-oxide (DMPO) was used as the probe for hydroxyl radicals,^(15)^ and 2,2,6,6-tetramethyl-4-piperidone hydrochloride (4-oxo-TEMP) was used for ^1^O_2_ detection.^(16)^ These probes have been applied previously in biological systems with successful detection of ROS.^(3,17)^

## Materials and Methods

### Oxidation of rhododendrol and rhododendrol-catechol in the presence of a spin-trap reagent for hydroxyl radical or an acceptor of singlet oxygen

Rhododendrol and rhododendrol-catechol were kindly provided from Kao Corporation (Tokyo, Japan). The oxidation of rhododendrol and rhododendrol-catechol in the presence of tyrosinase (from mushrooms; Sigma-Aldrich, Tokyo, Japan) was performed in the presence of a spin-trap reagent in a water bath at 37°C. A spin-trap reagent, DMPO (Labotech, Tokyo, Japan), was used to detect hydroxyl radicals and superoxide anions, while 4-oxo-TEMP (Sigma-Aldrich) was used for detecting ^1^O_2_. The appropriate reagent for detecting hydroxyl radical or ^1^O_2_ and rhododendrol or rhododendrol-catechol were dissolved in a 30 mM phosphate buffer (pH 7.4). Tyrosinase was added to this solution in an amount of 100 units (U)/ml to initiate the enzymatic oxidation reaction. The value of one enzyme unit of commercial tyrosinase was provided by the supplier, Sigma-Aldrich. Autoxidation of rhododendrol-catechol was carried out under the same reaction conditions described above except for the reaction in the absence of tyrosinase.

### Measurement of the absorption spectra of products formed during the oxidation of rhododendrol and rhododendrol-catechol with tyrosinase

The reaction mixture described above was filtered using a 0.22-µm syringe filter. The filtered reaction mixture was diluted 5 times with a 30-mM phosphate buffer (pH 7.4). The diluted reaction mixture was then transferred to a quartz cell (light path length of 1 cm). Its absorption spectrum for wavelengths of 200–800 nm was measured using a Multispec-1500 spectral measurement system (Shimadzu, Kyoto, Japan).

### Detection of hydroxyl radicals and ^1^O_2_ using ESR

 The reaction mixture was transferred to a quartz flat cell (150 µl) for ESR measurements. The optical path length was 0.25 mm. The ESR spectra of the various samples were recorded at room temperature using an X-band spectrometer (JES-FA-100, JEOL, Tokyo, Japan), which was operated at 9.43 GHz. The magnetic field was modulated at 100 kHz. The conditions for measurement of ESR spectra were as follows: microwave power of 4 mW; magnetic field of 335.5 ± 5.0 mT; field modulation width of 0.1 mT; sweep time of 2 min; time constant of 0.1 s.

The signal intensities were normalized with respect to a MnO marker, and the concentrations of the stable radical products were determined on the basis of the signal height using an external standard, 2,2,6,6-teteramethyl piperidinol (TEMPOL; Sigma-Aldrich).

### Estimation of bond dissociation energies of the oxygen-hydrogen bond in catechol derivatives

Catechol- and phenol-derivative models were constructed and optimized using density functional theory (DFT). The long-range and dispersion corrected ωB97X-D functional with 6-311G(d,p) basis sets were selected.^(18–20)^ It has been shown that ωB97X-D results in better estimates of bond dissociation energies.^(21)^ The optimized molecular structures were verified through vibration analysis. The equilibrium structures did not have imaginary frequencies. The total energies were corrected at the zero-point vibrational energy. The bond dissociation energy (BDE) was obtained using Equation (1):

*BDE* = *E*_ROH_ – *E*_RO_ (1)

where *E*_ROH_ is the total energy of each catechol- and phenol-derivative model, and *E*_RO_ is the total energy of each model in which hydrogen is dissociated, as estimated by a single-point calculation. All calculations were performed using the Gaussian 09 program.^(22)^

## Results and Discussion

### Hydroxyl radical generation during tyrosinase-catalyzed oxidation of rhododendrol and rododendrol-catechol

**Fig. 2:**
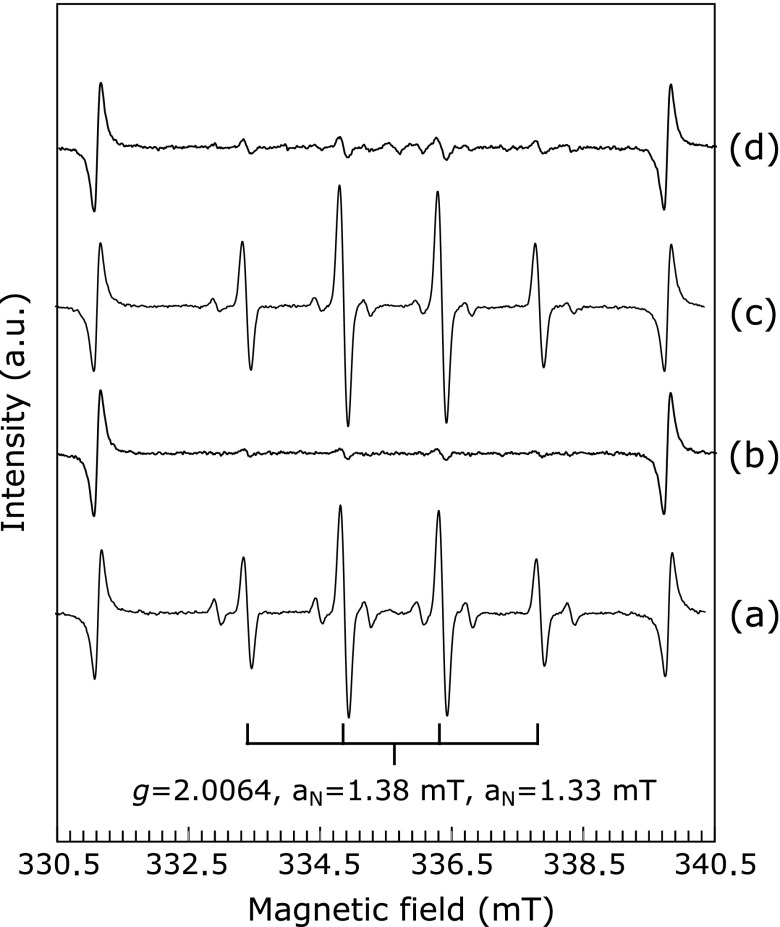
ESR signals of DMPO-OH during the oxidation of rhododendrol and rhododendrol-catechol with mushroom tyrosinase, and autoxidation of rhododendrol-catechol measured using 450 mM DMPO in a 30 mM phosphate buffer (pH 7.4): (a) 1 mM rhododendrol with 100 U ml^−1^ tyrosinase, (b) 1 mM rhododendrol, (c) 1 mM rhododendrol-catechol with 100 U ml^−1^ tyrosinase, and (d) 1 mM rhododendrol-catechol.

**Fig. 3:**
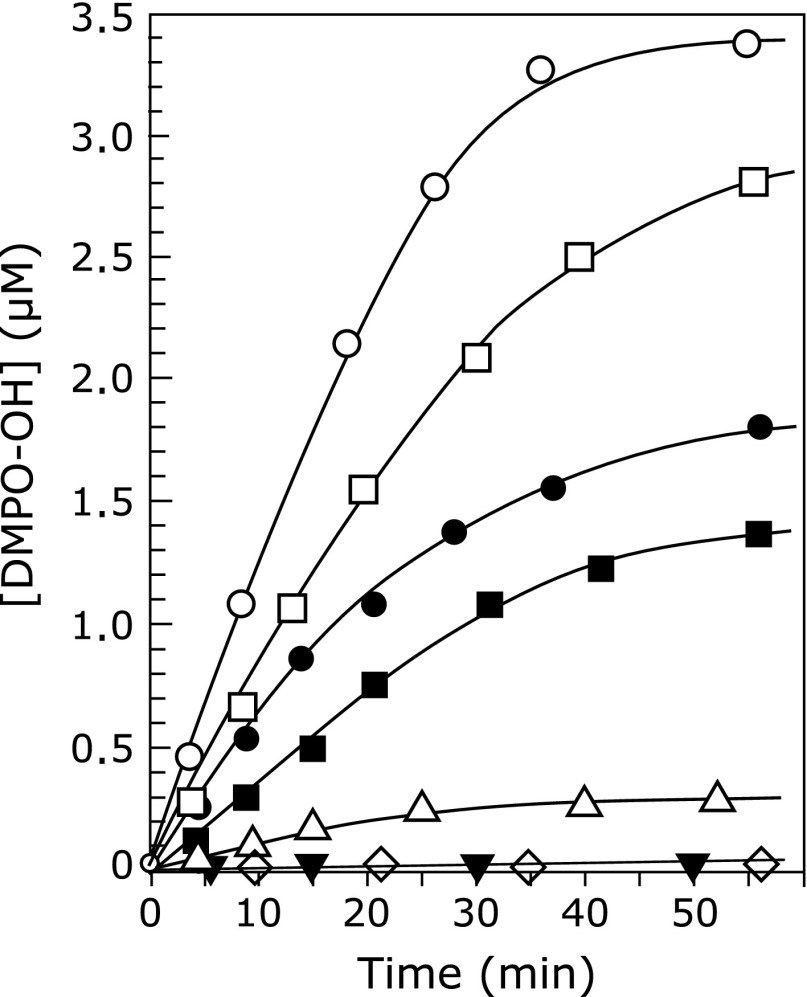
Time course of hydroxyl radical generation during the oxidation of rhododendrol, rhododendrol-catechol, l-tyrosine, and l-dopa with tyrosinase. Hydroxyl radicals were trapped by 450 mM DMPO: (○) 1 mM rhododendrol-catechol with 100 U ml^−1^ tyrosinase, (□) 1 mM rhododendrol with 100 U ml^−1^ tyrosinase, (I) 1 mM l-dopa with 100 U ml^−1^ tyrosinase, (▪) 1 mM l-tyrosine with 100 U ml^−1^ tyrosinase, (△) 1 mM rhododendrol-catechol, (◇) 1 mM rhododendrol, (▼) 1 mM l-dopa, (◆) 1 mM l-tyrosine.

 In previous work, rhododendrol and rhododendrol-catechol showed toxicity at a concentration of 1 mM in melanocytes having high tyrosinase activity.^(7)^ In order to estimate the amount of hydroxyl radical generated in tyrosinase-catalyzed oxidation of these two rhododendrol compounds, we performed the oxidation of 1 mM rhododendrol compound in the presence of mushroom tyrosinase, and measured ESR spectrum of spin adducts of DMPO with a hydroxyl radical (DMPO-OH). When 1 mM rhododendrol was added to the DMPO/tyrosinase solution, an ESR signal having a *g* value of 2.0064 and a hyperfine constant of 1.38 and 1.33 mT, attributable to DMPO-OH,^(15)^ was observed, as shown in Fig. 2(a). Without tyrosinase, hydroxyl radical generation was not detected (Fig. 2(b)). The amount of hydroxyl radicals increased rapidly for approximately 30 min (Fig. 3).

Hydroxyl radical generation was also observed in tyrosinase-catalyzed oxidation of rhododendrol-catechol (Fig. 2(c)). As shown in Fig. 3, the production rate of hydroxyl radicals in the rhododendrol-catechol oxidation was faster than those in the oxidation of l-tyrosine and l-dopa. The production rate was also faster than that in rhododendrol oxidation with tyrosinase. The higher toxicity of rhododendrol-catechol than rhododendrol may be caused by the difference in the amount of hydroxyl radicals generated.

DMPO can trap not only hydroxyl radical but superoxide anion. In the ESR spectra, the signal attributable to a spin adduct of DMPO with a superoxide anion (DMPO-OOH) was not observed. DMPO-OOH has a much shorter lifetime than DMPO-OH.^(23)^ Therefore, we were unable to determine whether superoxide anion was generated or not during our experiments.

During this period, the color of the reaction mixtures immediately changed from colorless to orange and pink, suggesting that rhododendrol is oxidized to other rhododendrol derivatives. According to the spectral change in the ultraviolet-visible (UV-vis) measurement of the reaction mixture, the absorbance around 300–600 nm increased immediately after the reaction started as previously reported.^(9)^ The increase in the absorbance indicates that rhododendrol quinone (Fig. 1A), cyclic rhododendrol quinone (Fig. 1B), and hydroxy rhododendrol-*p*-quinone (Fig. 1C) showing absorbance at approximately 400, 460, and 380 nm, respectively, are produced during this period. Therefore, hydroxyl radical occurs during conversion of rhododendrol to other quinone and catechol-derivatives initiated by tyrosinase.

Without tyrosinase, spectral changes for rhododendrol in phosphate buffer (pH 7.4) were not observed (data not shown), indicating that rhododendrol is not autoxidized at a pH of ~7. In addition, hydroxyl radical generation was not observed in the absence of tyrosinase. Thus, hydroxyl radical generation occurs only during tyrosinase-catalyzed rhododendrol oxidation.

Hydroxyl radical can be produced by reductive fission of hydrogen peroxide. As previously reported, hydrogen peroxide is produced during tyrosinase-catalyzed rhododendrol and rhododendrol-catechol oxidations.^(13)^ We also confirmed the hydrogen peroxide generation by measuring the increase in dissolved oxygen concentration by the addition of catalase. The hydrogen peroxide generated in the oxidation of rhododendrol and rhododendrol-catechol may cause hydroxyl radical generation. On the other hand, Tada *et al.*^(3)^ reported that hydroxyl radical generation via tyrosinase-catalyzed oxidation of l-tyrosine and l-dopa was not affected by the addition of catalase. Based on this result, they proposed that hydroxyl radical is produced via the decay of dicopper-peoxide intermediate formed by the reaction of oxygen molecule with the di-copper site of tyrosinase.^(3,24)^ Further investigations are required for elucidating the mechanism of hydroxyl radical generation in tyrosinase-catalyzed oxidation of rhododendrol and rhododendrol-catechol.

The quantity of hydroxyl radicals produced during the oxidation of rhododendrol and rhododendrol-catechol was higher than that during the oxidation of of l-tyrosine and l-dopa, as shown in Fig. 3, suggesting that hydroxyl radical generation by rhododendrol is abnormal compared with physiological melanin synthesis in melanocytes. The amount of hydroxyl radicals trapped by DMPO during tyrosinase-catalyzed oxidation of rhododendrol and rhododendrol-catechol was estimated to be approximately 1 µM for a reaction duration of 10 min as shown in Fig. 3. Riesz and co-workers reported that the efficiency of 200 mM DMPO to trap hydroxyl radical generated in gamma-irradiated aqueous solution was about 35%.^(25)^ Assuming that the efficiency in our experiment was same as the reported value, the actual amount of hydroxyl radical generated during the enzymatic oxidation can be estimated to be about 2.9 µM. This amount of hydroxyl radical is probably sufficient for exhibiting cell toxicity. According to the measurement of free radical generation in endothelial cell suspension (8 × 10^6^ cell ml^−1^) by exposing the cells upon air for 10 min, 0.3 µM of hydroxyl radical was generated and 90% of the cells was injured.^(26)^ Melanocyte may be more tolerant toward hydroxyl radical than endothelial cell due to quenching hydroxyl radical by melanin. Although tolerance of cells toward hydroxyl radical is dependent on the kind of cells, around 1.0 µM of hydroxyl radical generation is probably critical for cell viability. Further investigation regarding the correlation between the amount of hydroxyl radical generation in melanocyte and toxicity for the cell is ongoing.

### Hydroxyl radical generation during the autoxidation of rhododendrol-catechol

**Fig. 4:**
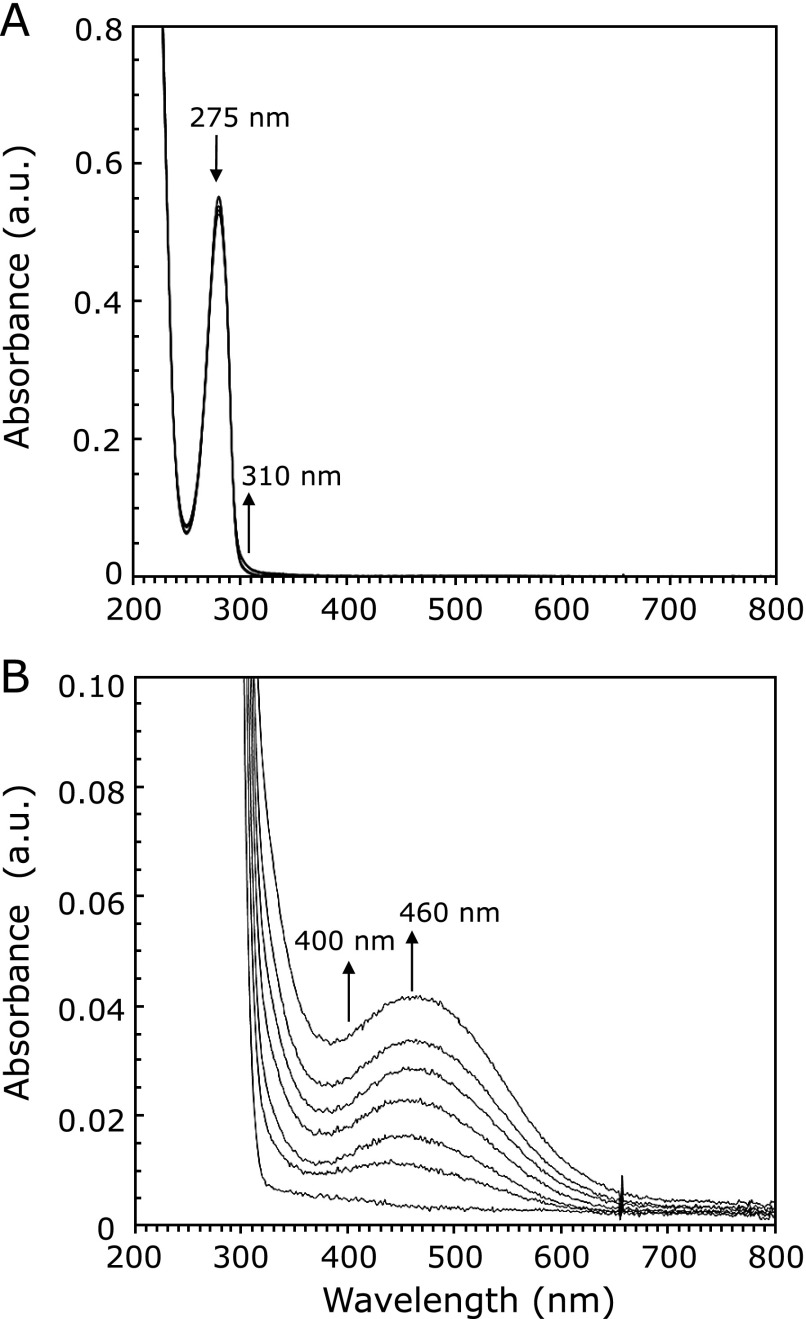
Spectral change during the autoxidation of rhododendrol-catechol. 1 mM rhododendrol-catechol in a 30 mM phosphate buffer (pH 7.4) was used for the reaction, and the reaction mixture was diluted 5 times with a 30 mM phosphate buffer (pH 7.4) for spectral measurement: (A) Spectra were measured at 20-min intervals from the start of the reaction until a period of 120 min, (B) Spectra shown in (A) are enlarged.

When rhododendrol-catechol was dissolved in a phosphate buffer (pH 7.4) in the presence of oxygen, the color of the solution gradually changed from colorless to pink. The spectral change of this solution is shown in Fig. 4. The absorbances around 460 nm and 300 nm, attributable to cyclic rhododendrol-quinone, increased gradually. During these spectral changes, the dissolved oxygen concentration decreased (data not shown). These results indicate that rhododendrol-catechol is autoxidized at a pH of 7.4. During the autoxidation, hydroxyl radical generation was observed in rhododendrol-catechol without tyrosinase (Fig. 2(d)), suggesting that rhododendrol-catechol shows toxicity even in cells having low tyrosinase activity.

Autoxidation mechanism of catechol includes the oxidation of the corresponding catecholate anions, which are in equilibrium with the parent catechols. The acidity dissociation constants (p*K*_a_) of rhododendrol and its derivatives are summarized in Table 1. p*K*_a_ of the phenol and catechol compounds listed in Table 1 were around 9, indicating that the phenol and catechol compounds occur in non-dissociated form at physiological pH. In that case, the bond dissociation energy of the oxygen-hydrogen bond in the phenol and catechol compounds can be used as an indicator for the occurrence of autoxidation because autoxidation is initiated by the homolytic cleavage of the oxygen-hydrogen bond of the non-dissociated form. Bond dissociation energies of catechol-derivatives estimated by DFT calculations are listed in Table 1. The bond dissociation energy of rhododendrol-catechol is lower than l-dopa, which is not autoxidized at a neutral pH, and is close to 6-hydroxy dopa and 6-hydroxy dopamine, which are easily autoxidized at a neutral pH. Thus, autoxidation of rhododendrol-catechol is related to its structure. Further, as shown in Table 1, bond dissociation energies of cyclic rhododendrol-catechol and hydroxyl rhododendrol-catechol were much lower than rhododendrol-catechol. Once rhododendrol-quinone is converted to rhododendrol-catechol, cyclic rhododendrol-catechol, or hydroxyl rhododendrol-catechol, autoxidation of these catechol derivatives occurs. Hydroxyl radical generation occurs during autoxidation. Autoxidation followed by hydroxyl radical generation may affect the toxicity of cells having low tyrosinase activity.

### ^1^O_2_ generation during oxidation of rhododendrol and rhododendrol-catechol

**Fig. 5:**
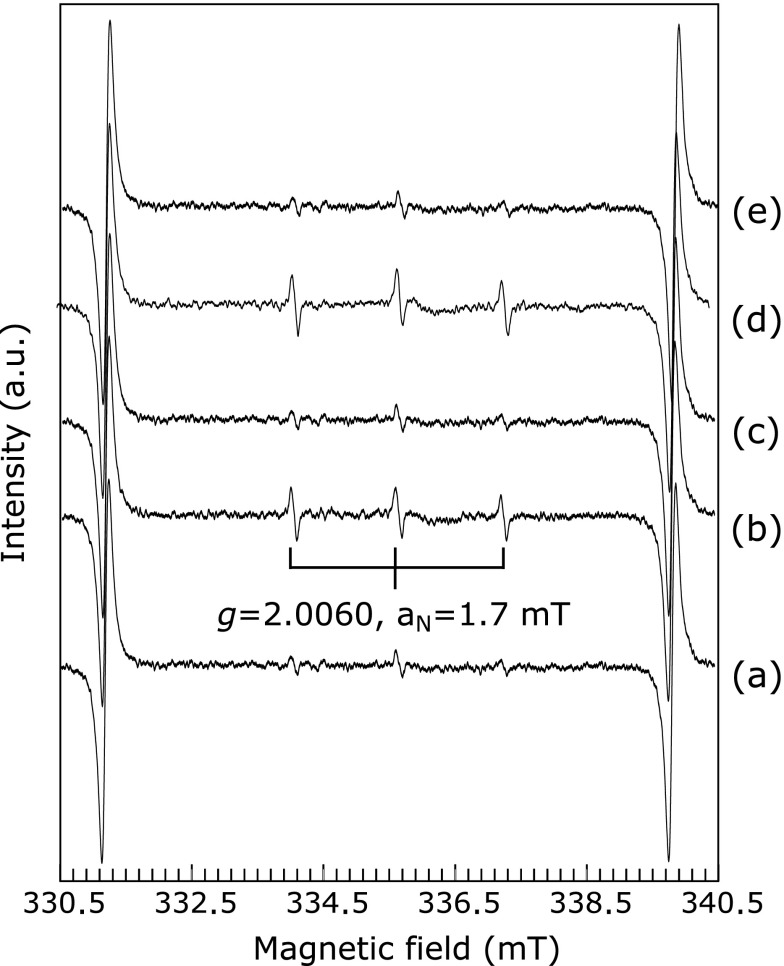
ESR signals of 4-oxo TEMPO during the oxidation of rhododendrol and rhododendrol-catechol with mushroom tyrosinase, and autoxidation of rhododendrol-catechol measured by using 50 mM 4-oxo-TEMP in a 30 mM phosphate buffer (pH 7.4): (a) No enzyme and no substrate, (b) 1 mM rhododendrol with 100 U ml^−1^ tyrosinase, (c) 1 mM rhododendrol, (d) 1 mM rhododendrol-catechol with 100 U ml^−1^ tyrosinase, and (e) 1 mM rhododendrol-catechol.

When ^1^O_2_ is reacted with 4-oxo TEMP, the nitroso radical is produced. This result corresponds to three equivalent ESR signals having a *g* value of 2.0054 and a hyperfine constant of 1.6 mT.^(16)^ As shown in Fig. 5(a), 4-oxo TEMP did not show a signal in a phosphate buffer (pH 7.4). In 1 mM rhododendrol with tyrosinase in a phosphate buffer (pH 7.4), an ESR signal attributable to 4-oxo TEMPO (*g* = 2.0060, a_N_ = 1.7 mT) was observed, as shown in Fig. 5(b). On the other hand, in the absence of tyrosinase, the ESR signal attributable to 4-oxo TEMPO was negligibly small, as shown in Fig. 5(c). The signal attributable to 4-oxo-TEMPO in tyrosinase-catalyzed rhododendrol oxidation was reduced when the oxidation reaction was performed in the presence of ^1^O_2_ quencher, histidine (25 mM). Therefore, the ESR signal of 4-oxo-TEMPO indicates ^1^O_2_ generation over rhododendrol oxidation initiated by tyrosinase.

**Fig. 6:**
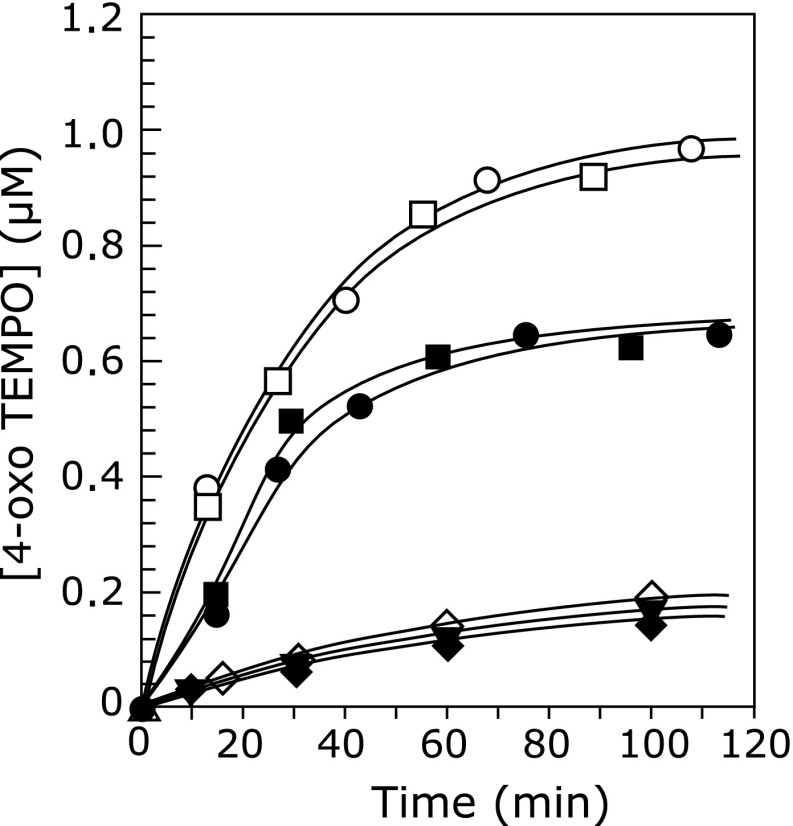
Time course of ^1^O_2_ generation during the oxidation of rhododendrol, rhododendrol-catechol, l-tyrosine, and l-dopa with tyrosinase. ^1^O_2_ was reacted with 50 mM 4-oxo-TEMP: (○) 1 mM rhododendrol-catechol with 100 U ml^−1^ tyrosinase, (□) 1 mM rhododendrol with 100 U ml^−1^ tyrosinase, (I) 1 mM l-dopa with with 100 U ml^−1^ tyrosinase, (▪) 1 mM l-tyrosine with 100 U ml^−1^ tyrosinase, (△) 1 mM rhododendrol-catechol, (◇) 1 mM rhododendrol, (▼) 1 mM l-dopa, (◆) 1 mM l-tyrosine.

The amount of ^1^O_2_ trapped by 4-oxo TEMP was estimated to be 0.9 µM for a reaction duration of 1 h, which is larger than that generated during tyrosinase-catalyzed oxidation of 1 mM l-tyrosine and l-dopa,^(27)^ as shown in Fig. 6. ^1^O_2_ generation was also observed in 1 mM rhododendrol-catechol with tyrosinease (Fig. 5(d)). On the other hand, ^1^O_2_ was not detected in autoxidation of rhododendrol-catechol, which may be caused by the slow oxidation rate.

The amounts of ^1^O_2_ generated via rhododendrol and rhododendrol-catechol oxidations with mushroom tyrosinase are probably lower than that of ^1^O_2_ exhibiting cell toxicity. According to the calculation of ^1^O_2_ generation in photodynamic therapy (PDT) previously reported, about 10^8^ ~ 10^9^ molecules of ^1^O_2_ per cell is required to reduce the surviving fraction of cells by 1/e.^(28,29)^ By assuming the diameter of a cell is 10 µm, volume of a cell can be calculated to be 5 × 10^−14^ L, and the concentration in a cell showing toxicity can be estimated to be 0.1–1.0 M. Compared to this amount, the amount of ^1^O_2_ produced during the oxidations of 1 mM of rhododendrol and rhododendrol-catechol with mushroom tyrosinase was much lower; approximately 10^−6^ M of ^1^O_2_ was produced for a reaction duration of 2 h as shown in Fig. 6. Rhododendrol and rhododendrol-catechol showed toxicity at a concentration of 1 mM in melanocyte having high tyrosinase activity.^(7)^ Therefore, ^1^O_2_ generated via rhododendrol and rhododendrol-catechol oxidations with tyrosinase does not cause cell toxicity.

Previous studies have reported that ^1^O_2_ are generated via hydroxyl radical during the oxidation of l-tyrosine and l-dopa with tyrosinase.^(27)^ Therefore, we performed the tyrosinase-catalyzed oxidation of rhododendrol and rhododendrol-catechol in the presence of both 4-oxo-TEMP and DMPO, resulting that the intensity of the signal for 4-oxo-TEMPO decreased. This result suggests that ^1^O_2_ generates via hydroxyl radical and/or superoxide anion in the the tyrosinase-catalyzed oxidation of rhododendrol and rhododendrol-catechol. Of the ^1^O_2_ generation proposed previously,^(30,31)^ the following reactions include hydroxyl radical and/or superoxide anion

^•^OH + O_2_^−^ → ^1^O_2_ + OH^−^  (1)

O_2_^−^ → ^1^O_2_ + e^−^  (2)

2O_2_^−^ + 2H^+^ → ^1^O_2_ + H_2_O_2_  (3)

^•^OH + ^3^O_2_ → [^•^OOOH] → ^1^O_2_ + ^•^OH  (4)

As mentioned above, hydrogen peroxides are generated during the oxidation of rhododendrol and rhododendrol-catechol. Therefore, the following reaction can be considered for the reaction of ^1^O_2_ generation.

H_2_O_2_ + HOO^−^ → ^1^O_2_ + H_2_O + OH^−^  (5)

These reactions are thermodynamically not favored. However, some of these reactions may occur at the active site of enzymes in cells. For instance, intermediate of oxygen activation at di-copper site of tyrosinase such as endoperoxide^(32)^ may be involved in the ^1^O_2_ generation.

## Conclusion

ESR signals attributable to hydroxyl radicals and ^1^O_2_ were observed during the tyrosinase-catalyzed oxidation of rhododendrol. Compared to tyrosinase-catalyzed oxidation of l-tyrosine, large amounts of hydroxyl radicals were generated during tyrosinase-catalyzed oxidation of rhododendrol. The amount of hydroxyl radicals generated increased with increasing tyrosinase activity. Further, ^1^O_2_ was also generated in the oxidation reaction. The amount of ^1^O_2_ generated in the tyrosinase-catalyzed oxidation of l-tyrosine was less than in that of rhododendrol. These results suggest that rhododendrol induces both hydroxyl radical and ^1^O_2_ generation in melanocytes. Melanin can react with ROS such as hydroxyl radicals and ^1^O_2_.^(33–36)^ Thus, melanocytes have the ability to protect themselves against hydroxyl radical and ^1^O_2_. Rhododendrol interferes with melanin synthesis because it competitively reacts at the active site of tyrosinase. Moreover, rhododendrol-catechol, the intermediate of rhododendrol oxidation in melanocyte, was autoxidized at a pH of approximately 7. At least hydroxyl radicals were generated during autoxidation. Therefore, generation of intermediates during tyrosinase-catalyzed oxidation of rhododendrol enhances oxidative stress in melanocytes. Thus, rhododendrol probably disrupts the balance between the antioxidant systems and ROS generation in melanocytes.

## Figures and Tables

**Table 1:** Bond dissociation energy of hydroxyl group in phenol and catechol compounds estimated by DFT calculations.

Phenol and catechol compound	p*K*_a_^a)^	Homolytic cleavage of O-H bond	Bond dissociation energy (kJ mol^−1^)
Rhododendrol	9.7	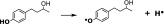	380
Rhododendrol-catechol	9.2	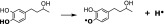	305
Cyclic rhododendrol-catechol	9.6	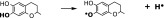	297
Hydroxy rhododendrol-catechol	9.1	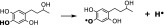	162
L-Tyrosine	9.3	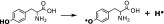	382
L-Dopa	8.7	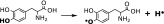	340 (pH = 7)
			281 (pH>8.7)
*tert*-Butylcatechol	9.2	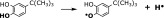	310
6-Hydroxydopamine	8.6	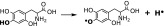	266
6-Hydroxydopa	8.7	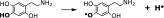	282

^a)^The acidity dissociation constants of a hydroxyl group in catechol or phenol moiety, which are calculated by ChemBioDraw Ultra 14.
